# Women’s experience of the health information process involving a digital information tool before commencing radiation therapy for breast cancer: a deductive interview study

**DOI:** 10.1186/s12913-023-09837-2

**Published:** 2023-08-09

**Authors:** Annika Grynne, Josefin Wångdahl, Sofi Fristedt, Frida Smith, Maria Browall

**Affiliations:** 1 Department of Nursing, School of Health and Welfare, Jönköping University, Jönköping, Sweden; 2 School of Research, School of Health and Welfare, Jönköping university, Jönköping, Sweden; 3 The Jönköping Academy for Improvement of Health and Welfare, School of Health and Welfare, Jönköping University, Jönköping, Sweden; 4 Aging Research Center, Department of Neurobiology, Care Sciences and Society, Karolinska Institutet and Stockholm University, Stockholm, Sweden; 5 Department of Rehabilitation, School of Health and Welfare, Jönköping University, Jönköping, Sweden; 6 Department of Health Sciences, Faculty of Medicine, Lund University, Lund, Sweden; 7 Regional Cancer Centre West, Gothenburg, Sweden; 8 Department of Technology Management and Economics, Chalmers University of Technology, Gothenburg, Sweden; 9 Dep of Oncology, Inst of Clinical Sciences, Sahlgrenska Academy, University of Gothenburg, Gothenburg, Sweden

**Keywords:** Breast cancer, Health information process, Digital information tool, Radiation therapy

## Abstract

**Background:**

Individuals undergoing radiation therapy for breast cancer frequently request information before, throughout and after the treatment as a means to reduce distress. Nevertheless, the provision of information to meet individuals needs from their level of health literacy is often overlooked. Thus, individuals information needs are often unmet, leading to reports of discontent. Internet and digital information technology has significantly augmented the available information and changed the way in which persons accesses and comprehends information. As health information is no longer explicitly obtained from healthcare professionals, it is essential to examine the sequences of the health information process in general, and in relation to health literacy. This paper reports on qualitative interviews, targeting women diagnosed with breast cancer who were given access to a health information technology tool, Digi-Do, before commencing radiation therapy, during, and after treatment.

**Methods:**

A qualitative research design, inspired by the integrated health literacy model, was chosen to enable critical reflection by the participating women. Semi-structured interviews were conducted with 15 women with access to a digital information tool, named Digi-Do, in addition to receiving standard information (oral and written) before commencing radiation therapy, during, and after treatment. A deductive thematic analysis process was conducted.

**Results:**

The results demonstrate how knowledge, competence, and motivation influence women’s experience of the health information process. Three main themes were found: Meeting interactive and personal needs by engaging with health information; Critical recognition of sources of information; and Capability to communicate comprehended health information. The findings reflect the women’s experience of the four competencies: to *access*, *understand*, *appraise*, and *apply*, essential elements of the health information process.

**Conclusions:**

We can conclude that there is a need for tailored digital information tools, such as the Digi-Do, to enable iterative *access* and use of reliable health information before, during and after the radiation therapy process. The Digi-Do can be seen as a valuable complement to the interpersonal communication with health care professionals, facilitating a better *understanding*, and enabling iterative *access* and use of reliable health information before, during and after the radiotherapy treatment. This enhances a sense of preparedness before treatment starts.

## Background

Health information is essential in preparing and supporting a person as an individual and in meeting their needs throughout different treatment procedures, such as the complex radiation therapy (RT) process [1, 2]. Furthermore, health information provision makes up a critical component of both person-centred care (PCC) and organizational health literacy (OHL) [3, 4]. PCC recognises a person’s capabilities, focusing on what is meaningful for them [5, 6] and their families [2]. Smith, Carlsson [7] illustrate that health information often outlines what the healthcare providers believe the person should know, rather than considering what the person needs in terms of appropriate information or best suited to their level of health literacy (HL). Thus, a person’s information demands are often unmet, leading to reports of discontent [2, 8–10]. Additionally, evidence shows that significant others also frequently experience such unfulfilled information needs [11]. Although significant others require tailored information to help them to cope with uncertainties and to provide support during the cancer trajectory, there is evidence to suggest that this is suboptimal in many instances [12, 13]. Within the scope of this article, referring to health information includes both clinical information and practical information. Clinical information relates to information about cancer, the RT process, and its side-effects, while practical information relates to parking, maps, important telephone numbers etc.

Breast cancer is the most widespread type of cancer amongst women [14, 15]. RT is currently the most common treatment to improve local control and overall survival for breast cancer [16]. While the RT procedure itself takes only a few minutes, it requires meticulous preparation, for example, to ensure correct positioning. Individuals within this patient group frequently request information throughout and after the RT treatment to reduce distress [17]. A coping strategy to manage this desire for information is to actively access cancer-related health information from various sources, such as healthcare professionals (HCPs), the Internet, significant others [18–20], and, more recently, digital information tools with Virtual-Reality (VR) technology [21]. The rapid growth of the Internet and innovative digital information tools has significantly influenced the proliferation of information available and changed the way in which women access and comprehend health information [22]. Individuals who previously accessed information as passive receivers now activly search for health information [23]. A recent study by Bender, Hueniken [24] found that 25% of the participants used the Internet to learn about cancer, while 21% did so to connect with other persons with similar diagnoses. Lee and Lin [25] suggest that reading health information online prior to attending clinical office visits may have a positive influence on the patient-HCP relationship. However, they also raise concerns regarding the challenges faced by patients to understand, appraise and apply the health information they access online. Thus, examining how individuals’ use a variation of information resources to access and comprehend health information prior commencing and undergoing RT seems justified.

As health information is no longer explicitly obtained from the HCPs, it is essential to examine the sequences of the health information process in general and in relation to HL. Understanding the specific characteristics of this process and individuals’ unique needs for information can provide HCPs with essential insight. This can enhance understanding for health information needs while safeguarding PCC in daily practice [26, 27]. HL is a concept seen as a health asset that encompasses crucial dimensions, each of which requires specific cognitive skills and depends on the quality of the information provided [28]. Both Liu, Wang [29] and Sørensen, Van den Broucke [28] state that, for a person to actively particpate in the health information process, they must believe in their own capability, understand the context and work in partnership with others. The integrated model of HL is framed within four competencies: *access*, *understand*, *appraise*, and *apply* within the health information process [28]. This model will be applied as the theoretical framework for this article. The model proposes that *access* comprises the ability to seek and obtain health information; *understand* includes the capability to comprehend the health information accessed; *appraise* refers the capacity to interpret, filter, and evaluate the health information that has been accessed; and *apply* describes the ability to communicate and use the information to make a decision to maintain and improve health [28]. Moreover, working towards best practice, OHL are designed to build a person-centred, evidence-based and quality-driven healthcare. As OHL has a responsibility to make it easier for all stakeholders to access, understand, appraise and apply health information [4] examining how this relates to the experience of the health information process is central. This paper reports on qualitative interviews of the experience of the health information process in women undergoing RT for breast cancer. This study is conducted as the qualtiative component of a larger study comprising 128 participants (intervention groups *n* = 59 and control group n = 52). The overal aim of the larger study was to evaluate a digital information tool targeting women diagnosed with breast cancer, before commencing RT, and during and after the treatment process [30].

The digital information tool, named Digi-Do tool was co-designed by the research group and key stakeholders. The description of the development process of the tool is presented elsewhere [31]. The tool consists of two separate mobile apps presenting health information in an innovative format. The first app encompassing VR-technology, enable the user a simulated visit to the RT department, experiencing the high-tech environment. Applying a VR-headset will support a sense of presence [32].

For the user who prefer not to use the VR-headset, the simulated visit can still be experienced by navigating through the environment, using their finger to navigate on their mobile-screen. The VR-app is designed to be operated in combination with an information app. The information app encompasses three sets of information in the form of evidence-based information (questions-and-answers), animated films, and practical information. Additionally, the person can easily share Digi-Do, allowing their significant others to access information [2, 33].

In today’s healthcare system patients are expected to take part and to be engaged in their own care. Consequently, to fully benefit and be able to actively participate to maintain and improve health they must be able to read and understand health instructions [34]. With many individuals struggling with health information the need for improved formats of communication and compelling health information is pressing.

Hence, to safeguard a PCC approach and HL responsive work while ensuring equality in health, gaining further knowledge of persons perceptions of the health information process is needed. The aim of this study is to explore how women diagnosed with breast cancer *access*, *understand*, *appraise*, and *apply* health information generally and specifically through a digital information tool before commencing a RT treatment, and during and after completion of the treatment.

## Method

### Study design

A qualitative research design, including semi-structured interviews, inspired by the integrated HL model [28], was chosen to enable critical reflection by the participating women [35].

### Setting and sample

The setting and recruitment of participants for the larger study comprised the surgical and the oncology departments in two hospitals in the Western part of Sweden. In the larger study the inclusion criteria was as follows; women aged 18 years or over who had a confirmed diagnosis of breast cancer and were due to commence RT and Neo-adjuvant treatments, endocrine therapies were accepted, the RT preparatory hospital visit occurred no earlier than one day post inclusion, were fluent in Swedish, had access to a smartphone, and had planned RT at either clinic enrolled in the study. In line with the study protocol of the larger study, using a blocked randomization method, the participants were randomised to either a control or an intervention group [30]. For the present qualitative study, only participants from the larger study’s intervention group were interviewed, as they were the only group with access to the Digi-Do, in addition to being given the standard information (oral and written). With the ambition to enable transferability and obtain heterogeneity, a strategic selection process was carried out, placing a focus on recruiting participants of a variety of ages and place of residence (Table 1).

**Table 1:** Descriptive statistics of participants (n = 15)

Characteristics	Number (%)
Age	
41–50	3 (20)
51–60	3 (20)
61–70	5 (33)
71–80	3 (20)
8–90	1 (1)
Mean, SD	63.47, 12.03
Marital status	
Married/ co-habiting	11(73)
Single	4 (27)
Employment	
Employed	6 (40)
Retiered	9 (60)
Residental area	
Within 50 km from the hospital	3 (20)
Within 50–250 km from the hospital	12 (80)
Co-morbidities Yes	8 (53)
Combination treatment Yes	12 (80)
DCIS Grade	
Grade 1	1 (1)
Grade 2	6 (40)
Grade 3	4 (27)
Invasive Cancer	3 (20)
Not listed	1 (1)
Treatment duration, days	
5 days	7 (47)
15 days	8 (53)

### Ethics approval

Permission to conduct the study was received from the management at the oncology, surgical, and RT departments before initiation of the larger study. Informed consent to partake in the qualitative study was obtained at the same time as informed consent was obtained for the larger study after explaining the outline, objectives, and content of the study. Each participant’s informed consent was reconfirmed verbally at the start of their interview. The study was performed in accordance with the medical research ethical standards of the Declaration of Helsinki. Ethics approval was obtained from the Swedish Ethical Review Authority (Dnr 2020 − 00170).

### Data collection

An integrated model of HL [28] was applied to provide an insight into the participants knowledge, motivation, and competence for actively engaging with the health information process. Individual, semi-structured qualitative interviews were held with 15 women between June 2021 and June 2022. The interview guide included topics [36] to explore how women experience the health information process aligned with breast cancer treatment and the RT treatment. The health information process comprises *access* to information from different sources; capability to *understand* and *appraise* accessed health information; and ability to *apply* the comprehended health information in relation to the RT treatment process, i.e., the adoption of the Digi-Do and sharing the Digi-Do with significant others. Additional interpretative questions were applied [36]. Three pilot interviews were conducted jointly by the first author and a senior researcher to test the interview guide and promote consistency throughout the interview process. The interview guide was deemed satisfactory in addressing the aim of the study and the pilot interviews were therefore included in the data analysis. The consecutive interviews were performed by the first author. Interview duration varied from 24 to 61 min, with a mean duration of 44 min. The interviews took place, in accordance with the women’s wishes, digitally via a video call (*n* = 9) or on the telephone (*n* = 3), or in person in their own home (*n* = 3). A consistent pattern of responses was recognised after having conducted 15 interviews, which indicated that data saturation had been reached, and this was deemed sufficient for the qualitative analyses of the study [37, 38]. All interviews were audio-recorded, and transcribed verbatim.

### Data analysis

A deductive thematic analysis process [39], based on the Integrated HL model [28], was conducted. Clarke and Braun [39] have outlined a guide through six phases of analysis, each phase equally crucial to ensure the rigor of the process, as shown in Table 2. Although the different phases for thematic analysis appear to be consecutive in order, the process of analysis and seeking out patterns is iterative and recursive in nature. Qualitative analyses were performed by the first author and regularly discussed and agreed by all co-authors. These discussions can be seen as being key to promoting reflexivity throughout the data analysis process as they enable alternative perspectives of the analysis process and findings to be completed [40].

**Table 2:** The six phases of thematic analysis [39]

Phase	Description of the process
1. Familiarisation	The transcribed data were read and re-read to ensure a familiarisation with the depth and breadth of the content. Simultaneously, a mind-map of ideas based on the four competencies developed.
2. Generating codes	The data were approached with the four competencies (*access*, *understand*, *appraise*, *apply*) in mind, used as codes to identify essential characteristics of the data. Within this phase a sense of the participants experience of the health information process and the Digi-Do was achieved.
3. Searching for themes	The codes were examined systematically and patterns of meaning relevant to the health information process were identified.
4. Reviewing themes	Identifying the ‘essence’ of each theme and subtheme, how they fit together. Discussions concerning potential overlaps and to ensure the themes reflected the data and the aim were undertaken. A thematic map was devised.
5. Defining and naming themes	A written detailed analysis was conducted for each individual theme to identify how the theme fit the overall statement of the data.
6. Producing the report	Preparing to write the article to tell the story of the data to confirm the merit and validity of the analysis. Data extracts capturing the essence of important points in the data were selected.

## Results

The results demonstrate how knowledge, competence, and motivation influence women’s experience of the health information process. Below we summarize these data in the context of three main themes: Harmonize interactive and personal needs engaging with health information; Critical recognition of sources of information; and Capability to communicate comprehended health information (Table 3).

**Table 3:** Themes and subthemes from qualitative data analysis

Meanings unit	Condensed unit	Code	Subtheme	Theme	Overall Theme
I used the information-app in my smartphone, I had a look every now and then.	Iterative access of information.	ACCESS & UNDER-STAND	*Different formats of information resources*	Harmonize interactive and personal needs engaging with health information	HEALTH INFORMATION PROCESS
In the first meeting with (the HCPs) you are not able to take in all the information.	Ability to take in information.		*Empowerment through tailored health information*		
… with google information, of course you pay attention to the sources.	Critical of online source.	APPRAISE	-	Critical recognition of sources of information	
Whenever someone has asked me, I have the app on my smartphone to show them what I have gone through.	Share and communicate using digital technology/ Digi-Do.	APPLY	*-*	Capability to communicate the attained health information	

### Harmonize interactive and personal needs engaging with health information

#### Different formats of information resources

During the waiting period before commencing RT, the women navigated through various sources of information. The assimilation of information using a combination of different media (Internet, interpersonal communication, the Digi-Do) simultaneously was described as enabling better comprehension of the RT treatment. The interpersonal meeting with the HCP at the surgical, oncology and RT departments was seen as being a valuable source of information and the health information provided there was deemed trustworthy. Nonetheless, at times, having a vast amount of information presented during a short space of time in the meetings with HCPs made it difficult for the women to comprehend the material. Further, the context influenced the conversation and the women’s capability to ask questions. Thus, to sit down in a consultation with an HCP was perceived as being more useful. The face-to-face meetings gave opportunities to ask questions and gain answers about their health from the experts. The meetings were reinforced with written information (brochures), which was often seen as helpful. Previous health experiences and expectations of the interpersonal meeting had an impact on understanding what had been discussed. It was also noted how the presence of a low emotional state could have a negative influence on the comprehension of the information. Additionally, side-effects from previous chemotherapy sessions could affect what the women remembered after the meeting.

*‘I can ask something, and it was the same when the doctors sat there and told me something and when I came out from there, I remembered maybe 10% of what had been said.’ (Participant #12)*

The Digi-Do was seen as being a valuable complement to the interpersonal meeting, enabling them to have quick access to reliable information. The tool enabled an increased understanding of their own health, cancer, and the RT process, both before, during and after the meeting. The participating women described how the Digi-Do presenting information in different formats (simulated visit, written text, voice-over, animated films) was seen as being a stimulating way to learn. The ability to choose what information to access, and to repeat and revisit the information, helped the women to comprehend and gain a better understanding of the meetings with HCPs and the RT process.

*‘It was probably a combination of seeing the environment and obtaining information … I was somehow more prepared for what would happen.’* (*Participant #3)*

The meetings with HCPs within the RT setting were perceived differently. Although the staff were portrayed as being friendly and professional, the environment was experienced as being stressful, with other patients waiting in line, leaving little or no time for the women to ask questions.

*‘It’s like in and out and in and out just like that*. So you don’t have many seconds to exchange words with them.’ (Participant #7)

Health-related barriers, such as reduced eyesight, made it difficult to read written text in brochures, on online websites, and in the Digi-do. Hence, the ability to listen to the voice-over in the apps facilitated comprehension of the information. Not common but observed was a negative attitude towards digital technology. A frustration linked to a lack of experience of using digital technology was expressed and there was an apprehension of making mistakes or damaging the equipment, for example, pressing the wrong key or when handling the VR-goggles.

Social media was seen as an effective medium for instantaneous communication with other people, despite a certain degree of suspicion around privacy. It was voiced how support in the style of emojis of hearts was encouraging, providing a sense of being cared for. To be active in breast cancer groups enabled contact with other women who shared similar experiences. This was seen to be beneficial as it reduced a sense of being alone.

*‘Had there not been social media and this had happened 15 years ago and there had been the pandemic at the same time, I think I would have experienced a greater sense of isolation.’ (Participant#1)*

#### Empowerment through tailored health information

The health information obtained from online sources, the Digi-Do, and, at times, the interpersonal meetings, was found to be too general. This was viewed as a disadvantage, as the women wished for information that they could relate to their own personal situation. Some of the women expressed how accessing information would only increase their distress. Consequently, they were restrictive in accessing information from both HCPs and other sources such as the Internet.

*’I told the doctors to only tell me only exactly what I needed to know.’ (Participant #12)*

For some of the women, the term breast cancer had negative associations, which at times led to adverse effects when searching online. Thus, negative associations hindered the women from knowing what to search for. Fear of not being sure of what type of information they would access, or what to do with the information, was expressed.

*‘I won’t become calm. To find out more about the cancer or treatment won’t contribute to my wellbeing. I just have to wait and see what is to come.’ (Participant #8)*

As most of the women had not previously attended the hospital where they received RT treatment, the ability to self-navigate where to go and what to see using the VR-application was very much appreciated. While some of the women enjoyed using the VR-goggles to gain a simulated view of the hospital setting, others experienced dizziness. At times there were some minor technical difficulties when handling the VR-goggles. The majority of the women preferred to navigate through the environment with their finger directly on the mobile screen as they found that they could still engage in walking through the environment without having to bother with the VR-goggles. The simulated visit to the high-technological environment with a voice-over explaining what they saw, for example, their position on the RT bed, was seen as a modern way to learn about the clinical setting. This was mentioned as being something that enabled them to better understand and made them feel less anxious.

*‘I have never experienced this kind of shifting pictures before, this VR-application where you can decide where to go and see where you are going to have RT was great!’ (Participant #7)*

Further, the flexibility to be able to access the Digi-Do using a smartphone was seen as beneficial. As many of the women often had their smartphones nearby, they could easily look at the set of questions-and-answers to obtain answers to any concerns that might come up.

*‘Sometimes a question pops up and you want to check it right away. So instead of having to go home and look up some paper that you have left somewhere you have the answer at hand straight away.’ (Participant #12)*

There was a voiced appreciation of having seen the environment before attending the first appointment. This facilitated a better understanding, which made the women feel more in control. It was also indicated that having previously seen the environment increased their sense of control and reduced distress, enabling the women to focus more of what was being communicated by the HCPs.

*‘… you can take in more of what is said during your first visit because you don’t have to be nervous or that you won’t be able to find where you are going.’ (Participant #10)*

The ability to share the tool, allowing significant others access to the information, was described as being valuable and beneficial, both for the women and for the person they shared the tool with. The VR-app in particular was shared with both children and elderly parents. It was found that sharing the tool with others enabled them to obtain a better understanding of the woman’s situation, which enhanced support. Further, it was expressed that just knowing that, for example, the spouse had seen and experienced what the women was going through enhanced a sense of alleviation for the women. Additionally, it was noted that the Digi-Do could be used as an aid to answer and clarify questions from concerned significant others.

*Using the tool, he (her husband) got an insight into it all, he gained access to the information and the simulated environment … it was fantastic, as he could be involved too.’ (Participant #4)*

The regularity and frequency of accessing information online or applying the Digi-Do varied. Like the use of Digi-Do, the rate of recurrence of seeking information on the Internet peaked during the waiting period for commencing RT and became less frequent once the treatment had started. With regards to the Digi-Do, it was found that the women primarily employed the VR-app before the start of the RT, while the information-app was utilized throughout the course of treatment. Some of the women re-visited the Digi-Do after completion of the RT treatment, as they found that this facilitated more of an understanding of all they had gone through.

### Critical recognition of sources of information

Information received from the HCPs was generally found to be presented in a straightforward language and was easy to interpret and appraise. In relation to information obtained on the Internet, most of the women saw themselves as being critical, reading only information on sites that they deemed credible. To be deemed credible, the Internet site had to be well-known, recommended by HCPs, and non-commercial. Others did mention reading information on sites without questioning the quality of the source. At times the information accessed on the Internet was validated with HCPs or significant others who worked within the medical profession. Accessing personal experiences shared on social media by other women diagnosed with breast cancer triggered mixed emotions. There was a voiced scepticism towards information accessed on social media groups as it at times was found to be biased and inaccurate.

*I feel that sometimes when you read blogs or similar you are dragged down and you become low. No, it’s nothing that suits me ...’ (Participant #11)*

Despite this, it was found how chatting with those sharing a similar experience was described as a relief and a way to affirm their frame of mind. The information attained in these conversations was interpreted and applied to their own situation, making this more credible rather than reading about it.

*‘… it is nice to talk to someone who knows what you’re talking about … someone who doesn’t get worried about me ... it’s nice to get confirmation that you are normal in your illness.’ (Participant #12)*

After initial apprehension, some women found that the Digi-Do was relevant in meeting their needs. They reported that the 360-degree images presented in the VR-application gave an accurate description of the environment. Nonetheless, it was voiced that the images in the VR-app were not always identical to the hospital space, due to recent refurbishment. The benefit of combining the different formats in the two applications was described as being useful, and the information was deemed to be both credible and easy to comprehend. One of the women, who worked in a clinical setting herself, thought that the information was of a satisfactory level and accurate.

*‘Even if I hadn’t known anything about healthcare, I probably would have perceived the Digi-Do as great … I thought the level of the information was satisfying.’ (Participant #8)*

### Capability to communicate the attained health information

Obtaining an understanding of the diagnosis and treatment process was essential for creating a sense of control. Further, this increased the sense of being prepared before starting RT. Having this knowledge encouraged the women to engage in conversations about health. Many of the women mentioned how their increased understanding enabled them to be more active in communicating with the HCPs. Their state of mind, being happy or sad, influenced the women’s capability of participating actively in decisions related to health.

*‘Then maybe I haven’t been really capable … I’ve been mostly sad and scared, maybe I haven’t taken advantage of engaging in communication with the health care professionals and participating in health decisions.’ (Participant #5)*

To experience a simulated visit to the clinical setting using the VR-application enabled a comprehension of the environment and the RT procedure. To see the environment before the initial visit enabled a sense of control. It was described how, by already being familiar with the hospital before their first visit, their distress was reduced as the women recognised where to go. As the women felt calmer, armed with this prior knowledge they were able to focus on different questions in the initial meeting with the HCP. Instead of most of the attention being placed on the high-technological environment and practical questions, it was suggested that the interpersonal meeting might be more focused on what was relevant to them as a person.

*‘… at the first visit you can take in more of what is being said because you don’t have to be nervous of not knowing where to go or about the environment.’ (Participant #10)*

It was found that the increased knowledge gained from accessing information through various sources enhanced the women’s ability to talk about health with other people, not just HCPs. As mentioned, having easy access to the applications on a smartphone enabled the women to show others what they had gone through in a simple and straightforward way, fuelling further interesting conversations.

## Discussion

This study, based on the integral HL model, facilitates an insight into women’s knowledge, motivation, and competence for actively engaging in the health information process. The findings reflect the women’s experience of the four competencies, *access*, *understand*, *appraise*, and *apply*, essential elements of the process. These competencies influence each other in a continuous process, where each is indispensable. The women in the study also *accessed* health information as a coping strategy to prepare, or to follow-up after interpersonal consultations. This finding corroborates prior research showing a link between knowledge and reduced anxiety [19, 25]. Despite placing a great degree of confidence in HCPs [41], it was found that there is a growing need for better *access* to reliable information on demand and the ability to repeatedly view information that might not be well-understood or remembered.

Digital information tool technology has rapidly evolved to address barriers to health information usage. Additionally, digital information tools have fundamentally transformed how persons *access* and *understand* health information. The Digi-Do, a digital information tool, co-designed with stakeholders, presents information in a format comprising a PCC-approach. The women considered the Digi-Do as a tool that allowed them to decide what information to *access* in their own time. It was found that the tool provided rapid dissemination of reliable information and enhanced a sense of preparedness for the RT during the waiting period. Further, it enhanced a sense of control in connection with the hospital visits as they were able to recognise where to go (within the hospital) and what to expect (of the RT treatment) by accessing the information gained through the simulated visits. In line with a PCC approach, it is essential to include significant others when such information is provided [2]. The Digi-Do offers a means for the women to share information with their significant others. The women in this study and their families appreciated this functionality. The ability to share the information was believed to be one of the main advantages of the Digi-Do and was of great importance for the women as it increased *understanding* and support from significant others. This is in line with Heynsbergh, Botti [42], who identified how digital technology has the potential to improve significant others’ access to information and support.

The most obvious benefit of the Digi-Do to the women was that the tool enabled flexibility and enhanced autonomy while facilitating a better *understanding* and knowledge of breast cancer and the RT treatment. There seemed to be a link between *understanding* information (HL) and psychological emotions such as anxiety (context) [43]. This confirms the relationship between context and HL. HL, as a dynamic proficiency, incorporates prior health knowledge and experience, health status, and cognitive abilities [44, 45]. As stated by Martensson and Hensing [46], a person’s level of HL might fluctuate depending on the emotional state of the person, the situation, and the environment. Thus, a prerequisite to meet the context-specific needs of persons with different levels of HL is that the digital information tool are co-designed with relevant stakeholders [47]. A better *understanding* about the RT process increased the women’s satisfaction before, during and after clinical treatment visits to the RT department. This result mirrors those of Fiksdal, Kumbamu [20], who performed focus group interviews to better understand online health-searching behaviours.

Balancing the need for and fear of information was described by the participating women. External factors, such as previous negative experience, made it difficult to *appraise* health information. The women had varied perspectives on *appraising* how the health information fit or did not fit their individual needs, which aligns with the integrated model of HL [28]. The competencies of the health information process incorporate the qualities of functional, interactive and critical HL [48], supporting the women to navigate through various information sources to maintain and improve health. *Appraisal* of health information is closely associated with the critical dimensions of HL. According to Nutbeam [48], critical HL illustrates the higher cognitive and social skills needed to enable to person to critically analyse information when determining whether to use and *apply* the information.

According to a recent scoping review comparing preferences for cancer health information sources, HCPs were seen as valuable sources, while the majority of participants still access information through the Internet [41]. The women in the present study described how a face-to-face consultation was perceived as an opportunity to *access* information, ask questions, and gain answers. While it was declared that the interpersonal meeting had a positive effect on *understanding*, challenges were also voiced. The women expressed that this could be forgetting what to ask during a consultation or that it at times was challenging to remember all the information. This can be related to the dynamics of HL and the effects of chemotherapy [49]. Thus, the Digi-Do functions to complement the face-to-face consultation rather than acting as a replacement.

Blödt, Kaiser [43] found that there seems to be a relation between satisfaction with the interpersonal meeting and the person’s relationship with the HCP. Our findings suggest that having increased knowledge of one’s own personal health and the RT treatment facilitated and advanced the interpersonal communication with HCPs. The importance for HCPs to meet people at their level of HL while applying a PCC approach is extensively described in the literature [26, 29]. Additionally, the VR-technology enabled the participants to familiarise themselves with the environment prior to the meeting. This increased their sense of control and reduced distress while encouraging them to *apply* their obtained knowledge, thereby facilitating an interpersonal meeting with the HCPs where the information had a focus on and was relevant to the woman as a person instead of focusing only on the high-technological environment. The experience of the health information process is influenced by personal, situational, and societal determinants [28]. It is vital that health organisations, striving to be OHL, have the capacity to meet the needs of all patients including those with potential health related barriers. Practicing PCC focusing on the needs of the person does not always require face-to-face interaction [50] but digital information technologies can facilitate support of individuals’ need of information at their level of HL. Additionally, presenting information in different formats enable the user to experience simulated environments, while read or listen to the information.

A strength of the present study is its focus on participant´s experience of the health information process in general and through the Digi-Do before commencing RT. Connecting HL to health outcomes, the integrated model of HL represents the impact of societal, personal and situational factors on HL [28]. Applying the integrated HL model in the deductive analysis of the qualitative data may have limited the scope of the analysis. At the same time, using this model applying a deductive approach was found to enhance the focus on the iterative phases, that are characteristic of the health information process [28], while enabling a better understanding of participants experience of the health information process. The semi-structured interviews were conducted at the same time for all participating women (one month post completion of RT). This allowed for a credible description of the experience of the health information process before, during and after RT treatment and reduced the risk of recall bias. The location of the interviews varied. Despite being in line with the women’s wishes, this disparity may have influenced the quality of the data collection and each interview situation. However, the processes and discussions amongst the research team during the phase of data analysis helped to minimize research bias and ensure rigor. Although we are aware that the small sample size of this qualitative data makes it problematic to draw any general conclusions, we see a strength in it being a two-center study. We therefore believe that this supports the reliability of the findings and that new insights are easily transferable across settings. Including predominantly white, Swedish speaking women only can be seen as limiting the generalizability of the results. Conversely, as some of the RT processes are universal, as is the high-technological environment, we consider that it is possible that persons diagnosed with other types of cancer due to commence RT would also benefit by using the Digi-Do. The first author had little previous experience of qualitative interviews, which may be seen as a limitation in relation to important nuances that could have been overlooked. However, it can be argued that the initial three interviews, conducted together with a senior researcher, ensured credibility of the whole interview process [51]. This study complements some of what is already know about the health information process. We would like to suggest that it goes one step further by providing useful evidence for the need for tailored digital information tool innovations such as the Digi-Do to enable an iterative health information process, before, during and after the RT process. The advancement of digital information technology has improved breast cancer care by providing interactive tools. Proving to be a valuable complement to the meeting with HCPs is not single-handed enough for successful implementation for a digital information tool. Future research should focus on longitudinal studies to better understand the potential of digital information tools on communication outcomes, personal interaction as well as reducing distress in individuals undergoing RT for breast cancer.

## Conclusion

The findings formulate evidence of the need for tailored digital information tool innovations such as the Digi-Do to enable iterative *access* and use of reliable health information before, during and after the RT process. Applying Digi-Do as a complement to the interpersonal communication with HCPs had a positive impact on the women’s *understanding* of their cancer diagnosis and the RT treatment. This enhanced a sense of preparedness before the treatment started. The findings from this study highlight the benefits of the Digi-Do in enabling *access* to different formats of information to promote *understanding*, encourage the *appraising* of the information provided, and, finally, to *apply* this knowledge to maintain and promote health. Having knowledge and understanding of a person’s experience of the health information process will place HCPs in a unique position to communicate information that is appropriate to a woman’s level of HL, identifying information needs that are relevant to them while safeguarding PCC.

## Data Availability

The dataset used during the current study available from the corresponding author on reasonable request.

## Abbreviations

DIT: Digital Information Tool
HCPs: Health Care Professionals
HL: Health Literacy
OHL: Organizational Health Literacy
PPC: Person-Centred Care
RT: Radiation Therapy
VR: Virtual Reality
